# Attachment insecurity and breadcrumbing engagement in young adults: a cross-sectional, cross-country study in India and Spain

**DOI:** 10.1186/s40359-023-01404-y

**Published:** 2023-10-25

**Authors:** Vivek Khattar, Nuria Huete, Raúl Navarro

**Affiliations:** 1 Faculty of Education & Psychology, The Maharaja Sayajirao University of Baroda, Vadodara, 390002 India; 2 Faculty of Business Economic, University of Castilla-La Mancha, Albacete, 02071 Spain; 3 Faculty of Education and Humanities, University of Castilla-La Mancha, Cuenca, 16071 Spain

**Keywords:** Breadcrumbing, Attachment insecurity, Anxious attachment, Avoidant attachment, Cross-country research; Young adults

## Abstract

**Background:**

Breadcrumbing, defined as the act of sending out flirtatious, but non-committal text messages to lure a sexual/romantic partner without expending much effort, has gained attention in popular culture and the media due to its relevance to contemporary dating dynamics. However, there is lack of evidence of the association between attachment insecurity and breadcrumbing This study aims to uncover the potential relationship between breadcrumbing engagement and attachment insecurity among Indian and Spanish young adults.

**Methods:**

Data were collected through an anonymous online survey answered by 334 adults in India and by 348 adults in Spain aged between 18 and 40 years old. A linear regression model in both countries was run to examine breadcrumbing engagement and its relationship with the set of sociodemographic variables included in the study (participants’ age and sex, sexual orientation, relationship status and educational level) and the two dimensions of attachment insecurity (anxious and avoidant).

**Results:**

The results showed that insecure attachment, both anxious and avoidant, were associated with engaging in breadcrumbing in both the countries. However, there was a stronger association between anxious attachment and breadcrumbing in India compared to Spain, where the association was stronger between avoidant attachment and breadcrumbing.

**Conclusions:**

Findings from this study offer insight into an under-studied practice in the context of interpersonal relationships (i.e., breadcrumbing behaviour) and show the importance of attachment theoretical framework to hypothesize and analyse expectations regarding strategies to negotiate intimate relationships and the breadcrumbing experience particularly.

## Background

Dating and romantic relationships have experienced a remarkable transformation with the advent and growing popularity of social networking sites and dating applications [1]. Online platforms have not only expanded the pool of potential partners [2, 3], but they have also reshaped the dynamics of dating, making more common behaviours such as ghosting, orbiting, and breadcrumbing [4–6].

Breadcrumbing has gained attention in popular culture and the media due to its relevance to contemporary dating dynamics. It has been the subject of discussion in multiple articles, blogs, and television shows, as it describes a common experience that many people have gone through in modern relationships [7–9]. Breadcrumbing has been defined as “the act of sending out flirtatious, but non-committal text messages in order to lure a sexual/romantic partner without expending much effort” [10] and “sporadically sending someone flirtatious yet non-committal text messages or random social media “likes” to keep the person’s dating expectations of a possible relationship going, although the sender has no actual intentions of dating” [11]. The term breadcrumbing derives from the metaphor of following the trail of breadcrumbs left by someone to mark a path, like the tale of Hansel and Gretel [12]. Thus, the breadcrumber leaves little clues or signs of interest, such as occasional text messages, superficial flirtation, or sporadic invitations to go out, but without any real commitment or significant emotional investment [13]. More recently, breadcrumbing has been described as subtle manipulation tactic in dating relationships where the perpetrator “displays love, affection and sexual interest to their partners at the beginning of dating, often posing to be charming, flirtatious, and attractive” [14], but finally evolving into rather incongruent behaviour characterized by lack of commitment and avoidance of interactions.

Breadcrumbies receiving these mixed signals may build up expectations and hopes thinking there is genuine interest, only to find that the interest quickly disappears or decreases [6]. The lack of clarity and commitment in the actions of the breadcrumbing perpetrator can create an emotionally uncertain and destabilizing environment [6, 14]. Although there is still little empirical evidence regarding the breadcrumbing phenomenon, the available research has shown that experiencing breadcrumbing is associated with feelings of helplessness and self-doubt, lower self-esteem, experience of loneliness, and is also related with lower life satisfaction and difficulties in the development of future relationships [14, 15]. Breadcrumbing has been also associated with problems on self-concept, self-confidence and emotional stability on those who engage in it [16].

### Variables associated with breadcrumbing engagement

Limited research has been carried out to analyse factors associated with engaging in breadcrumbing behaviours. Available evidence has shown that the increased use of online dating apps, engaging in short-term relationships, and practicing online surveillance is related with breadcrumbing [13]. Despite a general lack of data, some studies have highlighted the absence of significant gender differences in engaging in breadcrumbing [13, 16]. However, qualitative research available on receivers’ experiences of breadcrumbing found more difficulties to obtain the data on men’s experiences in comparison to women’s experiences [14]. Therefore, more data is needed before suggesting the gender differences in breadcrumbing, and this study aimed to further analyse if there are gender differences in breadcrumbing involvement. The answer to the following questions will be sought in accordance with this purpose: Is there any gender difference in breadcrumbing engagement? Due to a paucity of research in this regard, the analysis of gender differences was exploratory in nature, and no concrete hypotheses were posed.

With regard to age, available studies have shown that breadcrumbing behaviours are common in adolescents’ and young adults’ populations [14, 16], with emerging adults (18–25 years old) reporting slightly higher scores on breadcrumbing behaviours [13]. This could be in part because emerging adults are learning how to navigate the emotionally complex field of intimate relationships which includes the interpersonal communication skills and personal responsibilities. It allows them to use harmful strategies to negotiate their romantic relationships [17, 18]. This study also aimed to further explore potential age differences answering the following research question: Is there any differences in breadcrumbing engagement between emerging adults and young adults? Considering past research, it could be expected that breadcrumbing involvement will be higher among emerging adults compared to young adults.

Beyond sociodemographic variables, popular media have associated several factors to breadcrumbing, such as insecure attachment, narcissistic personality, dysfunctional strategies to manage romantic relationships, fear of commitment, attention desire, self-esteem problems and emotional issues [19, 20]. However, these associations have not been empirically analysed. Therefore, research is needed to truly understand what psychological and social factors are associated with breadcrumbing. At this time, there is only one study examining one of the factors associated with breadcrumbing. Concretely, Willis et al. [21] investigated whether Dark Triad traits predicted breadcrumbing behaviours among young adults. They found that those who had breadcrumbed someone reported significantly higher vulnerable narcissism and Machiavellianism views. Yet, there is lack of evidence of the association between attachment insecurity and breadcrumbing. The purpose of the present study was to examine the relationships between breadcrumbing and attachment insecurity styles in those who engage in it.

### Attachment insecurity and breadcrumbing

Attachment theory describes how early bonding experiences with caregivers correlate with the way that adults interact in their emotional relationships in adaptative or maladaptive ways [22–24]. Research has shown that negative parent-child interactions in childhood, such as overprotection, psychological abuse, and neglect, as well as authoritarian and rejecting parenting, are related to adult attachment insecurity. Attachment insecurity is understood as individuals’ maladaptive patterns of relating to romantic partners [25].

Attachment insecurity includes two dimensions: anxiety and avoidance. Anxiety refers to the degree in which an individual is concerned that others will not be supportive when needed and anxiously requests love and support. Avoidance refers to the degree in which an individual suspects others’ intentions and defensively attempts to preserve social and emotional autonomy [26]. Different outcomes have been found in relational and mating contexts according to these two dimensions. For example, past research has found that anxiously, but not avoidantly, attached individuals are more motivated to use online dating and initiate more friendship, romantic and sexual relationships with other dating apps users [27]. People with anxious attachment values intimacy and are highly interested in romantic relationships. However, they may fear abandonment and rejection, and constantly seek validation from their partner, all of which can be fulfilled to some extent by online dating [25]. On the contrary, people with avoidant attachment tend to avoid emotional intimacy in romantic relationships [28]. They may find close relationships to be distressing and be reluctant to display warmth toward others [29] or engage in online dating [30]. In consequence, they may maintain emotional distance, value independence, be distrustful toward partners and prone to sabotage close relationships before they get too close [31, 32]. Avoidant attachment has also been associated with dissociating sex from romantic feelings [33] whereas anxious attachment has been associated with pursuing sex to achieve closeness with partners [31].

Although it is a controversial statement that men lean toward avoidant attachment formation and women lean toward anxious attachment formation [34], there is some supportive evidence to corroborate these findings. For example, Simpson et al. [35] reported that men scored higher on the dismissing style, and women scored higher on the preoccupied style. However, Schmitt et al. [36] found that men do not necessarily have a more dismissive form of attachment than women across all cultures; it may depend on sociocultural indicators. Regarding age differences, Segal et al. [37] found that younger adults experience anxious attachment more frequently than older adults. Similar findings were reported by Chopik et al. [38], indicating that attachment anxiety was more commonly reported in younger adults compared to middle-aged and older adults, while attachment avoidance was less commonly reported in younger adults compared to middle-aged and older adults. Even though the primary goal of this study was to examine the relationship between breadcrumbing engagement and attachment insecurity, we have also included gender and age as additional variables.

In relation to the associations between breadcrumbing and attachment insecurity, there are no studies analysing these relationships. However, previous research has found a strong relationship between attachment insecurity and the “hard-to-get” (HtG) behaviour, a practice with a resemblance to breadcrumbing. Playing HtG has been described as a practice during the dating process where “someone restricts demonstrations of interest in potential dating partners as a way of making oneself more desirable” [39]. Bowen & Gillath [40] examined the association between attachment insecurity and HtG behaviors and found that avoidant attachment was related to playing HtG, whereas anxious attachment was associated with the pursuit of HtG others. They concluded that the use of HtG may serve mating goals and preferences for more insecure individuals.

Based on these findings, attachment insecurity is also likely to play a role in breadcrumbing. Past research has shown that individuals with secure attachment are more likely to have more satisfying romantic relationships and relational outcomes compared to individuals with insecure attachment [41]. To this regard, Khattar et al. [14] explained that people with secure attachment are more engaged in relationships involving intimacy compared to people with avoidant attachment. Following this assumption, they hypothesised that, in the context of breadcrumbing behaviours, it is likely that individuals who engage in it will tend to have an avoidant attachment orientation to satisfy their need for attention without becoming emotionally involved. On the contrary, individuals with an anxious attachment style will be more exposed to experience breadcrumbing, as they have a greater need for emotional closeness and may be attracted to the superficial interaction of breadcrumbers to fulfil this need. Subsequently, individuals with an anxious attachment style will be less likely to engage in breadcrumbing behaviours compared to individuals with an avoidant attachment style.

However, research has not yet explored these relationships. Therefore, this study aimed to examine whether there is an association between attachment insecurity styles and engagement in breadcrumbing. The answer to the following question will be sought in accordance with this purpose: How does anxious and avoidant attachment correlate to breadcrumbing engagement? Considering previous research on the relationships between attachment insecurity styles and romantic relationships, we could expect that individuals with avoidant attachment, who are usually reluctant to commit and seek closeness, will have a positive correlation with breadcrumbing involvement. On the other hand, individuals with anxious attachment, who are generally inclined to commit in order to fulfil their need for emotional closeness and social belongingness, will likely exhibit a negative relationship with breadcrumbing engagement.

### The role of culture

The cultural environment has a deep impact on a wide range of social behaviours, including attachment [42, 43]. For example, countries founded in interdependence understandings of the self (eastern cultures) where individuals tend to be more dependent on others and more fearful of rejection, may promote more anxious attachment. Countries founded in independence understandings of the self (western cultures) where autonomy and personal goals are valued overt that of the collective, may promote more avoidant attachment [42, 44, 45]. Indeed, research has shown that cultures characterized by collectivism report higher anxious attachment [36], whereas cultures characterized by individualism report higher avoidant attachment [46].

Culture is an important variable that can also influence breadcrumbing through socialized values, expectations, attitudes and norms toward intimacy and romantic relationships [14]. For example, countries where commitment and exclusivity in dating relationships is prioritized may discourage behaviours such as breadcrumbing; whereas countries that embrace casual or non-committal relationships may have a higher tolerance for breadcrumbing.

These potential relationships are examined across young adults in India and Spain to study whether there are differences in the hypothesized associations across these two samples. India is considered a more collectivistic country than Spain [47]. This suggest that Spain places more emphasis on individual autonomy, personal achievement, and self-expression, while Indian culture values interdependence, group harmony, and loyalty. Moreover, Spain tends to have a relatively looser cultural orientation, while India has a tighter cultural orientation [48, 49]. Looser cultures tolerate a broader range of behaviour and are more permissive, whereas tighter cultures have stricter norms and greater conformity to social rules. In tight societies, such as India, young adults, despite their growing independence, appear to have less autonomy compared to Western societies, where greater freedom is granted in making choices concerning relationships, including their formation and termination, with a large number of non-committal relationships [50, 51]. In the current study, we have selected these two countries for their different cultural characteristics.

However, there are no studies examining and comparing breadcrumbing behaviours from countries with diverse cultural backgrounds. As such, the ways in which breadcrumbing is rooted in culture remains unclear. This study aimed to analyse the potential relationship between breadcrumbing and attachment insecurity (anxious and avoidant) among Indian and Spanish young adults. The answer to the following question will be sought in accordance with this purpose: Does the relationship between insecure attachment styles and breadcrumbing differs between countries? We anticipate that cultural factors may contribute to differences in the relationship between attachment insecurity styles and breadcrumbing. However, given the exploratory nature of the study, we do not propose any specific direction for the potential differences.

### Current study

The aim of the present study was to determine whether there is a relationship between insecure attachment and breadcrumbing engagement. We used cross-sectional data gathered from two sample of Indian and Spanish young adults.

In summary, our guiding questions and hypotheses are as follows: [1] Due to mixed and limited findings on gender differences in breadcrumbing, we cannot specify a hypothesis but are instead guided by the question of whether there are gender differences in breadcrumbing engagement [2]. Consistent with prior research, we expect emerging adults to be more engaged in breadcrumbing [3]. Based on previous findings regarding attachment insecurity and patterns of relating to romantic partners, we expect that avoidant attachment will be positively related to breadcrumbing engagement, whereas anxious attachment will be negatively related to breadcrumbing engagement [4]. Lastly, we explore if cultural factors play a role in shaping the relationship between attachment insecurity styles and breadcrumbing. Additionally, we investigate whether differences in terms of gender or age are the same in the India sample as in the Spanish sample.

## Methods

### Participants sampling procedure

Participants recruitment was made by snowball sampling method. Data were collected through an anonymous online survey distributed via social media, WhatsApp, and email. Inclusion criteria were: [1] aged between 18 and 40 years old; [2] being able to fill online surveys; [3] Indian and Spanish nationality. The number of participants was determined with G*Power (version 3.1.9.7), based on an a priori power analysis. A conservative mean effect size f2 of 0.15, power of 0.95, alpha of 0.05, and a maximum of 12 predictors were assumed, suggesting a minimum required size of 123 cases in each country. The survey was answered by 334 adults in India and by 348 adults in Spain aged between 18 and 40 years old. An instructional manipulation check (IMC) was used to verify that the participants had read the survey instructions. Based on Oppenheimer, Meyvis, and Davidenko [52], the IMC consisted in two additional items included within the measure’s items, which were designed similarly to the other items in terms of length and response format. However, unlike the regular items, the IMC items required participants to disregard the standard response format and, instead, provide a confirmation that they had read the instruction. Thirty participants in India and fourteen participants in Spain failed this check and were removed before running analyses.

The final sample in India was made up of 304 participants aged 18 to 40 years (mean age = 22.17 years; SD = 3.47). The sample was 81.3% female (n = 247), 17.1% male (n = 52) and 1.6% non-binary or other gender (n = 4). Moreover, 87.9% stated being heterosexual, 4.9% bisexual and 7.2% were lesbian or gay. In Spain, the final sample was made up of 334 participants aged 18 to 40 years (mean age = 28.02 years; SD = 5.51). The sample was 51.2% female (n = 171), 47,3% male (n = 158) and 1.5% non-binary or other gender (n = 5). Moreover, 73.7% stated being heterosexual, 16.8% bisexual and 9.6% were lesbian or gay. Details of the demographic characteristics of the participants in both countries are shown in Table 1.

**Table 1:** Sociodemographic characteristics by country

	India (n = 304)		Spain (n = 334)	
Variable	n	%	n	%
Age (M ± SD)	22.17 ± 3.47		28.02 ± 5.51	
Emerging adults (18–25 years)	232	76.3%	118	35.3%
Young adults (26–40 years)	72	23.7%	216	64.7%
Gender				
Men	52	17.1%	158	47.3%
Women	247	81.3%	171	51.2%
Non-binary/other gender	4	1.6%	5	1.5%
Sexual orientation				
Heterosexual	269	87.9%	246	73.7%
Non-heterosexual	37	12.1%	88	26.3%
In a committed relationship				
No	175	57.6%	129	38.6%
Yes	129	42.4%	205	61.4%
University-level education achieved				
No	207	68.1%	125	37.4
Yes	97	31.9%	209	62.6%

### Measures

#### The breadcrumbing in affective-sexual relationships questionnaire

(BREAD-ASR; [16] is a 16-item measure that assesses breadcrumbing engagement behaviours. Item examples: “I avoid being in person with my partner”; “I contact my partner when I feel alone”; “Communication with my partner depends on my interest and availability”. Participants indicate on a 5-point Liker scale from 1 (totally disagree) to 5 (totally agree) their levels of agreement/disagreement with the statements. Higher levels of breadcrumbing engagement are represented by a higher total score. Scale mean was computed by averaging the responses to all the 16 items. The BREAD-ASR showed a satisfactory content and construct validity with an internal consistency (Cronbach´s Alpha) of 0.83 [16]. The confirmatory analysis (CFA) in the current sample indicated a good fit in the measurement models for both countries: India (KMO test = 0.931; Bartlett sphericity test, χ² S − B = 120; (p < .001); NFI = 0.849, CFI = 0.884; CMIN/DF = 3.66; RMSEA = 0.074), and Cronbach’s alpha of 0.916; Spain (KMO test = 0.58; Barlett sphericity test, χ² S − B = 273 (p < .001); NFI = 0.77, CFI = 0.819; CMIN/DF = 3.9; RMSEA = 0.078) and a Cronbach’s alpha of 0.825. In addition to Cronbach’s alpha, McDonald’s omega (ω) was calculated and the results were acceptable to both countries (Spain: ω = 0.817; India: ω = 0.923).

#### The experiences in close relationships Scale-revised questionnaire

(ECR-RD8; [53] is an eight-item measure for evaluating adult attachment. The scale is comprised of two four-item subscales: anxiety and avoidance. Each item is rated on a 7-point Likert scale ranging from 1 (*strongly disagree*) to 7 (*strongly agree*). The ECR-RD8 showed acceptable model fit according to majority of criteria and internal consistency (McDonald’s Omega) was 0.83 for anxiety and 0.82 for avoidance in previous studies [53]. The confirmatory analysis (CFA) in the current sample indicated a good fit in the measurement models for both countries: India (Anxiety scale: KMO test = 0.806; Bartlett sphericity test, χ² S − B = 579.440 (p < .001); NFI = 0.806, CFI = 0.955; CMIN/DF = 3.45; RMSEA = 0.08; *α* = 0.865; ω = 0.867. Avoidance scale: KMO test = 0.706; Bartlett sphericity test, χ² S − B = 230.186 (p < .001); NFI = 0.854, CFI = 0.921; CMIN/DF = 3.95; RMSEA = 0.0809; *α* = 0.699; ω = 0.695); Spain (Anxiety scale: KMO test = 0. 806; Bartlett sphericity test, χ² S − B = 593.106 (p < .001); NFI = 0.938, CFI = 0.955; CMIN/DF = 3.45; RMSEA = 0.08; *α* = 0.855; ω = 0.858. Avoidance scale: KMO test = 0.673; Bartlett sphericity test, χ² S − B = 252.076 (p < .001); NFI = 0.873, CFI = 0.945; CMIN/DF = 3.98; RMSEA = 0.0813, *α* = 0.604; ω = 0.625).

#### Control variables

Participants provided information about their age, self-identified gender, sexual orientation, relationships status and educational level. All these variables were recoded into binary variables: age (1 = 18–25 years, 2 = 26–40 years), self-reported gender (1 = women and non-binary, 2 = men), sexual orientation (1 = heterosexual, 2 = non heterosexual); educational level (1 = no university level achieved; 2 = university level achieved); relationship status (1 = non-committed relationship, 2 = committed relationship), and were controlled for to account for possible effects.

### Procedure

Data were collected between May 1 and August 10, 2022. The study was conducted in compliance with the ethical standards of APA as well as following the 1964 Declaration of Helsinki, its later amendments, and comparable ethical standards. The study protocol was reviewed and approved by the Social Research Ethics Committee (SREC) of the University of Castilla-La Mancha (Approval Number. CEIS-646,931-R2H8). All participants were informed about the purpose of the study, as well as their anonymous and voluntary participation. Before answering the survey questions, participants gave their informed consent online. Participants did not receive any rewards for their participation.

### Data analysis

Firstly, descriptive statistics and correlation analysis were conducted. Secondly, regression analyses were conducted to examine the associations between attachment insecurity and breadcrumbing behaviour. As the dependent variable in the study was continuous, a linear regression model in both countries was run to examine breadcrumbing behaviour and its relationship with the set of sociodemographic variables included in the study (participants’ age and sex, sexual orientation, relationship status and educational level) and the two dimensions of attachment insecurity (anxious and avoidant). The analyses were carried out using the SPSS 28.0 statistical software package.

## Results

### Descriptive statistics and correlations

The main descriptive statistics for the variables included in the study are presented in Table 2. The breadcrumbing mean score was less than two in Spain and more than two in India. Considering that the response range was from 0 to 4, these were moderated scores in both countries. Statistically significant differences were found between countries, with higher scores in India (*M* = 2.44, *SD* = 0.89) compared to scores in Spain (*M* = 1.93, *SD* = 0.57), although with a small effect size (t(638) = 9.93, p < .001, *d* = 0.07). With respect to the dimensions of attachment insecurity, the mean scores for anxious attachment were significantly higher in India (*M* = 2.99, *SD* = 1.24) compared to scores in Spain (*M* = 2.37, *SD* = 1.10), although with a small effect size (t(638) = 6.68, p < .001, *d* = 0.07). Mean scores for avoidant attachment were similar in India (*M* = 1.96, *SD* = 0.81) and Spain (*M* = 1.90, *SD* = 0.82) and significant differences were not found (t(638) = 0.97, p = .33).

**Table 2:** Descriptive statistics and results of correlational analysis of variables

	India (n = 304)		Spain (n = 334)				
	M	SD	M	SD	1	2	3
1. Breadcrumbing	2.44	0.89	1.93	0.57		0.665**	0.252**
2. Attachment anxiety	2.99	1.24	2.37	1.10	0.289**		0.174**
3. Attachment avoidance	1.96	0.81	1.90	0.82	0.513**	0.190**	

**p < .01

Note: correlations for the Indian sample are above the diagonal, those for the Spanish sample are below the diagonal

Based on the Pearson correlation matrix, positive relationships were found between breadcrumbing and attachment insecurity in both countries. However, the relationship between breadcrumbing and anxious attachment was stronger in India (*r* (304) = 0.665, *χ*^2^ = 0.44) compared to Spain (*r* (334) = 0.289, *χ*^2^ = 0.08). On the contrary, the relationship between breadcrumbing and avoidant attachment was stronger in Spain (*r* (334) = 0.513, *χ*^2^ = 0.26) compared to India (*r* (304) = 0.252, *χ*^2^ = 0.06).

Due to the higher participation of women in the sample from both countries, an ANOVA analysis was conducted, using gender as a variable to test for significant differences concerning the studied variables in both countries. The data shows that when analysing breadcrumbing engagement, gender reaches significance in Spain (F(4,330) = 6.30, *p* < .001) but not in India (F(4,300) = 0.516, *p* = .724). Gender also reaches significance in avoidant attachment in Spain (F(4,330) = 2.93, *p* = .013) but not in India (F(4,330) = 0.845, *p* = .498). Regarding anxious attachment, gender does not reach significance in either India (F(4,300) = 0.323, *p* = .323) or Spain (F(4,330) = 0.858, *p* = .509).

### Results of regression analysis

Sociodemographic variables included age, gender, sexual orientation, and educational level were inputted together in Step (1) Relationship status was inputted in Step (2) In Step 3, the two dimensions of attachment insecurity (anxious and avoidant) were inputted. Sociodemographic variables and relationships status were introduced as dichotomous variables whereas anxious and avoidant attachment were introduced as continuous variables. The results of Steps 1–3 of the hierarchical regression analyses are reported in Table 3.

**Table 3:** Multiple regression analyses to examine the associations between breadcrumbing and attachment insecurity

	India (n = 304)			Spain (n = 334)		
Variables	*B*	*SE*_*B*_	*β*	*B*	*SE*_*B*_	*β*
Step 1						
Age	0.24	0.13	0.11	-0.03	0.006	-0.03
Gender	-0.10	0.13	-0.04	0.32	0.06	0.29***
Sexual Orientation	0.11	0.15	0.04	0.13	0.07	0.11
Educational level	-0.18	0.12	-0.09	0.11	0.06	0.09
	*R*^2^ (Adj. R^2^) = 0.016 (0.003) *F* = 1.223			*R*^2^ (Adj. R^2^) = 0.098 (0.086) *F* = 7.979***		
Step 2						
Age	0.38	0.12	0.18**	-0.002	0.006	-0.02
Gender	-0.18	0.12	-0.08	0.25	0.06	0.23***
Sexual Orientation	0.12	0.14	0.04	0.12	0.07	0.09
Educational level	-0.08	0.11	-0.04	0.10	0.06	0.09*
Relationship status	-0.73	0.09	-0.40***	-0.19	0.07	-0.15**
	*R*^2^ (Adj. R^2^) = 0.173 (0.159) *F* = 12.491***			*R*^2^ (Adj. R^2^) = 0.120 (0.105) *F* = 7.951***		
Step 3						
Age	0.22	0.09	0.10*	-0.002	0.005	-0.018
Gender	-0.06	0.09	-0.03	0.14	0.06	0.12*
Sexual Orientation	-0.05	0.11	-0.02	0.06	0.06	0.05
Educational level	-0.02	0.08	-0.01	0.14	0.05	0.12*
Relationship status	-0.39	0.08	-0.21***	-0.05	0.06	-0.04
Anxiety attachment	0.42	0.03	0.58***	0.08	0.02	0.17***
Avoidance attachment	0.10	0.04	0.09*	0.32	0.03	0.46***
	*R*^2^ (Adj. R^2^) = 0.505 (0.493) *F* = 43.101***			*R*^2^ (Adj. R^2^) = 0.368 (0.353) *F* = 24.124***		

Note: Gender (1 = women, 2 = men); Age (1 = 18–25 years, 2 = 26–40 years); Sexual Orientation (1 = heterosexual, 2 = non heterosexual); Educational level (1 = no university level achieved; 2 = university level achieved); Relationship status (1 = non-committed relationship, 2 = committed relationship)

*p < .05; **p < .01; ***p < .001

The results presented for Step 1 did not show any significant effect of sociodemographic variables on breadcrumbing engagement in the Indian sample. However, the results revealed a significant main association of gender and breadcrumbing engagement in the Spanish sample (*B* = 0.32, *SE*_*B*_ = 0.06, *β* = 0.29, *p* < .001). That is, in comparison to women, men were more likely to report higher levels of breadcrumbing. The results presented for Step 2 revealed that, for the Indian sample, age was significant associated with breadcrumbing (*B* = 0.38, *SE*_*B*_ = 0.12, *β* = 0.18, *p* = .002). Breadcrumbing seems to be higher among young adults (26–40 years). Moreover, the relationships status was associated with breadcrumbing in both India (*B* = − 0.73, *SE*_*B*_ = 0.09, *β* = − 0.40, *p* < .001) and Spain (*B* = − 0.19, *SE*_*B*_ = 0.07, *β* = − 0.15, *p* = .008). That is, breadcrumbing seems to be higher among adults in non-committed relationships. Additionally, in this second step gender remained significantly associated with breadcrumbing in Spain, and educational level was also significantly associated with breadcrumbing (*B* = 0.10, *SE*_*B*_ = 0.06, *β* = 0.09, *p* = .012). Breadcrumbing engagement seems to be higher among Spanish participants with university educational level achieved. However, the explanatory power of this second model is weak, with 15% of the variance explained in India, and the 10% in Spain.

The results of Step 3 showed that associated variables in Step 2 remained significant in both countries, except for relationship status in Spain. More importantly, results revealed that anxious attachment was significant and positively associated with breadcrumbing in both India (*B* = 0.42, *SE*_*B*_ = 0.03, *β* = 0.58, *p* < .001) and Spain (*B* = 0.08, *SE*_*B*_ = 0.02, *β* = 0.17, *p* < .001). In the same line, avoidant attachment was significantly and positively associated with breadcrumbing in both India (*B* = 0.10, *SE*_*B*_ = 0.04, *β* = 0.09, *p* = .027) and Spain (*B* = 0.32, *SE*_*B*_ = 0.03, *β* = 0.46, *p* < .001). Breadcrumbing seems to be strongly associated with insecure attachment dimensions. However, there are differences between the two countries in the type of the attachment insecurity more related with breadcrumbing. While in India the relationship is stronger with anxious attachment, in Spain it is stronger with avoidant attachment. The explanatory power of this third model is acceptable, with 49% of the variance explained in India, and the 35% in Spain.

## Discussion

This study was conceived to test the relationship between breadcrumbing and attachment insecurity among young adults in India and Spain, controlling for sociodemographic variables and relationship status. After the inclusion of attachment insecurity, significative associations between sociodemographic variables and breadcrumbing engagement differs between the two countries, although the correlations found are moderate to low. In India, age and relationship status were correlated to breadcrumbing, which indicates that young adults who were in the non-committal relationship were more likely to be engage in breadcrumbing. In Spain, correlations were found between gender, educational level and breadcrumbing engagement which indicated that men with university educational level were more like to be engage in breadcrumbing. However, since correlations were not found in the two country samples and the correlations found in each country were low, it is not possible to conclude clear age and gender differences.

Beyond sociodemographic variables, the results showed that insecure attachment, both anxious and avoidant, were associated with engaging in breadcrumbing in both the countries. The results of this study are consistent with related research showing that low anxious or avoidant attachment styles are associated with more positive romantic relationships and relational outcomes [41], and are in line with research indicating that individuals with attachment insecurity are less likely to be involved in healthy close relationships involving intimacy [14].

Results confirm our hypothesis regarding the positive relationships between avoidance attachment and breadcrumbing. Our findings suggest that avoidant attachment might pose a risk for being involved in breadcrumbing. Individuals with avoidant attachment style tend to create relational distance because they feel uncomfortable with interpersonal intimacy, suppress their feeling in social interactions, and are self-reliant [42, 54]. This way, breadcrumbing may be employed as a behavioural strategy to preserve their social an emotional autonomy, avoiding the distress of getting close with others. This is consistent with related research showing that individual with avoidant attachment are more prone to sabotage close relationships, and maintain independence and emotional distance from others [31, 32]. It is also in line with studies showing that avoidant attachment is related to behaviours sharing some similarities with breadcrumbing such as playing HtG, a strategy used to avoid emotional closeness withing the mating context [40].

For the hypothesis regarding the relationship between breadcrumbing and attachment insecurity, the results were different than expected. It was posited that there will be no positive relationship between breadcrumbing engagement and anxious attachment style, as individuals with anxious attachment are more prone to initiate friendships, romantic and sexual relationships, looking for intimacy and emotional closeness [25]. This behaviour propensity might lead to seeking validation from their partners, get carried away by compliments and hopes, and even build dependency, making them susceptible to behaviours such as HtG [40]. However, our findings suggest that anxious attachment is also related to being involved in breadcrumbing. Despite the unexpectedness of this relationship, this result is in line with research showing that attachment anxiety is significantly associated with perpetration of in-person and cyber psychological abuse [55].

One possible explanation for the relationship between anxious attachment and breadcrumbing behaviour is that individuals with anxious attachment often crave validation and reassurance from their partners. In some cases, breadcrumbing can be a manipulative tactic used to gain attention from the significant one [21]. This way, by alternating moments of connection followed by periods of silence or withdrawal, they leave the other person in a state of uncertainty and confusion, hoping that the other person will chase after them, thus validating their worth and importance. Another possible explanation is related with the fact that individuals with anxious attachment are ambivalent in their relational tendencies [56]. Individuals with anxious attachment may oscillate between seeking closeness and intimacy (known as the pull behaviour) but also pushing their relationship partners away fearing rejection or feeling insecure (known as the pushed behaviour). The intermittent and inconsistent nature of breadcrumbing can be a strategy used within this push-and-pull cycle in the relationship. Further research is needed to address how relational goals and motivations for using breadcrumbing are related to insecure attachment (both anxious and avoidant).

Finally, it is interesting to note that there was a stronger association between anxious attachment and breadcrumbing in India compared to Spain, where the association was stronger between avoidant attachment and breadcrumbing behaviour. One of the reasons could be related to the different understandings of the self, where India holds an interdependence approach and Spain holds an independent approach [57]. Therefore, anxious attachment is more likely associated with collectivistic cultures like India, and avoidant attachment is more likely associated with more individualistic cultures like Spain [46]. This also has an impact on relational goals and how people in each country manage their close relationships, which has a direct link to understanding differences in breadcrumbing behaviours. However, our study offers cross-country, rather than cross‐cultural comparisons. Future research must be conducted with participants from a wider range of countries that differ in their understandings of self to better examine cross-cultural differences in the associations between attachment insecurity and breadcrumbing behaviour.

### Limitations

A number of limitations should also be noted when interpreting the findings of this study. First, although the sample comprised by a similar number of adults in both countries, distribution of participants in terms of age and gender differed between the two countries, so findings are likely to somewhat be biased and generalizations should be made with caution. This difference distribution probably explains the differential associations found by country regarding the relationships between age, gender, and breadcrumbing. Future research should avoid the imbalance of gender and age, and also analyse the relationships explores in younger and older samples. Additionally, meta-analytic reviews have emphasized the importance of relationship duration as a crucial factor moderating the association between adult attachment and satisfaction/commitment in romantic relationships [58]. Future studies should not only consider the participants’ relationship status (as the present study did) but also consider the duration of their relationships. This will help to examine whether the association between attachment insecurity and breadcrumbing differs according to the length of committed relationships.

Second, data was collected by self-report short scales to avoid fatigue, but self-report measurements are subject to social desirability that may compromise the validity of the associations between the variables examined. Moreover, although breadcrumbing was measured with a validated scale covering various aspects (e.g., sporadic communication patterns, mixed signals, and the lack of commitment), a degree of uncertainty will always exist when dealing with the measurement of complex psychological/behavioural phenomena, such as breadcrumbing. While researchers work carefully to ensure construct validity, it is challenging to create a scale that captures every aspect of a construct without any overlap with other related constructs. Future studies should aim to establish a clear and comprehensive conceptualization of breadcrumbing. This involves identifying its core features, distinguishing it from related constructs (e.g., benching), and specifying the different forms it may take in various interpersonal contexts. As our understanding of breadcrumbing phenomena deepens, it may necessitate revising or refining the measurement scales accordingly.

Third, another limitation regards to the fact that we have only explored breadcrumbers’ perspective. The exclusive analysis on breadcrumbing others does not allow to exhibit the complexity of breadcrumbing and limit the analysis of its relationship with attachment insecurity. However, the lack of an establish scale to measure breadcrumbing victimization limited possible comparisons between breadcrumbers’ and breadcrumbies’ perspectives. Future research should develop a reliable instrument to measure breadcrumbies experiences to cover the different roles in the breadcrumbing phenomena and, additionally, to analyse if breadcrumbers are also breadcrumbies, as it occurs in other behaviours such as ghosting [59].

Fourth, data was gathered through snowball sampling which creates challenges in terms of representation and randomisation [60]. Moreover, the cross-sectional nature of the study makes not possible to determine the causality of the associations. Finally, the study did not analyse the relationships between breadcrumbing and other personal variables, such as personality traits, empathy or sociosexual orientations [61]. Future studies should focus on these variables and also social and cultural variables such as values and tolerant attitudes toward abuse [62].

### Practical implications

Our findings may also have practical implications. First, the topic is an important subject to explore affective-sexual relationships that are initiated/maintained through dating apps. Although the current study doesn’t particularly focus on online dating, this behaviour pattern can be observed in relationships that are primarily formed through online platforms. Therefore, this study can become a future scope for research taking into account variables like different dating platforms, sexuality, personality factors, etc. Additionally, it can be used to understand dynamics other than romantic relationships – for example, employer-employee, parent-child and teacher-student relationship.

Second, the study of breadcrumbing unfolds a certain relational dynamic that might be helpful for psychologists to understand a set of behavioural tendencies. In this sense, research can equip psychotherapists to formulate individual and couples therapy framework in several ways, for example, [1] identifying clients’ attachment patterns and explore how these patterns impact their behaviour in relationships, [2] helping clients to establish and maintaining healthy boundaries in their relationships, reducing the risk for breadcrumbing or it potential effects, [3] support clients in processing breadcrumbing and developing resilience to face dating challenges, and [4] guiding clients towards rebuilding trust and developing secure attachment bonds.

Finally, this study can be utilized by dating and marriage apps to modify their algorithm. By gaining insights into attachment styles and communication patterns, and their connection to breadcrumbing behaviours, dating apps could have the opportunity to personalize user experiences. They can achieve this by suggesting potential matches based on attachment compatibility, thereby helping to foster healthier connections.

## Conclusion

This study analysed the association of attachment insecurity (anxious and avoidant) and breadcrumbing in young adults from two different countries: India and Spain. Observed associations between the two variables show that these two phenomena are related. Insecure attachment might put a risk for breadcrumbing behaviour and an obstacle to establish healthy close relationships. Despite the limitations mentioned, findings from this study offer insight into an under-studied practice in the context of interpersonal relationships (i.e., breadcrumbing) and show the importance of attachment theoretical framework to hypothesize and analyse expectations regarding strategies to negotiate intimate relationships and the breadcrumbing experience particularly.

## Data Availability

The datasets used and/or analysed during the current study available from the corresponding author on reasonable request.

## List of abbreviations

BREAD-ASR: Breadcrumbing in Affective-Sexual Relationships Questionnaire
HtG: “hard-to-get” behaviour
IMC: Instructional Manipulation Check
ECR-RD8: Experiences in Close Relationships Scale-Revised questionnaire
